# Clarification

**Published:** 2007-12

In the November Focus article [“Carbon Capture and Storage: Blue-Sky Technology or Just Blowing Smoke? Environ Health Perspect 115:A538–A545 (2007)], Jeff Chapman, chief executive officer of the Carbon Capture and Storage Association, suggested €62 billion as the potential annual, not cumulative, revenue from the European carbon dioxide cap-and-trade system.

